# Optical Monitoring of the Magnetization Switching of Single Synthetic-Antiferromagnetic Nanoplatelets with Perpendicular Magnetic Anisotropy

**DOI:** 10.1021/acsphotonics.3c00123

**Published:** 2023-04-28

**Authors:** S. Adhikari, J. Li, Y. Wang, L. Ruijs, J. Liu, B. Koopmans, M. Orrit, R. Lavrijsen

**Affiliations:** † Department of Applied Physics, Eindhoven University of Technology, P.O. Box 513, 5600 MB Eindhoven, Netherlands; ‡ Institute for Complex Molecular Systems, Eindhoven University of Technology, P.O. Box 513, 5600 MB Eindhoven, The Netherlands; § Huygens-Kamerlingh Onnes Laboratory, LION, 2300 RA Leiden, Netherlands; ∥ School of Mechatronics Engineering, Harbin Institute of Technology, Harbin 150001, P. R. China

**Keywords:** photothermal microscopy, magnetic circular dichroism, magneto-optical Kerr effect, single-particle imaging, nanoparticles, chirality, nanophotonics

## Abstract

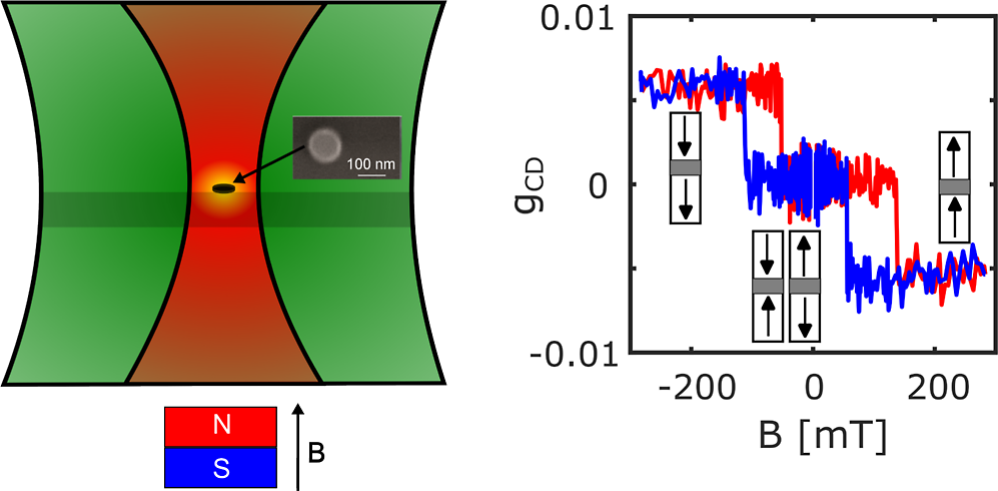

Synthetic antiferromagnetic nanoplatelets (NPs) with a large perpendicular magnetic anisotropy (SAF-PMA NPs) have a large potential in future local mechanical torque-transfer applications for e.g., biomedicine. However, the mechanisms of magnetization switching of these structures at the nanoscale are not well understood. Here, we have used a simple and relatively fast single-particle optical technique that goes beyond the diffraction limit to measure photothermal magnetic circular dichroism (PT MCD). This allows us to study the magnetization switching as a function of applied magnetic field of single 122 nm diameter SAF-PMA NPs with a thickness of 15 nm. We extract and discuss the differences between the switching field distributions of large ensembles of NPs and of single NPs. In particular, single-particle PT MCD allows us to address the spatial and temporal heterogeneity of the magnetic switching fields of the NPs at the single-particle level. We expect this new insight to help understand better the dynamic torque transfer, e.g., in biomedical and microfluidic applications.

## Introduction

**Figure 1:**
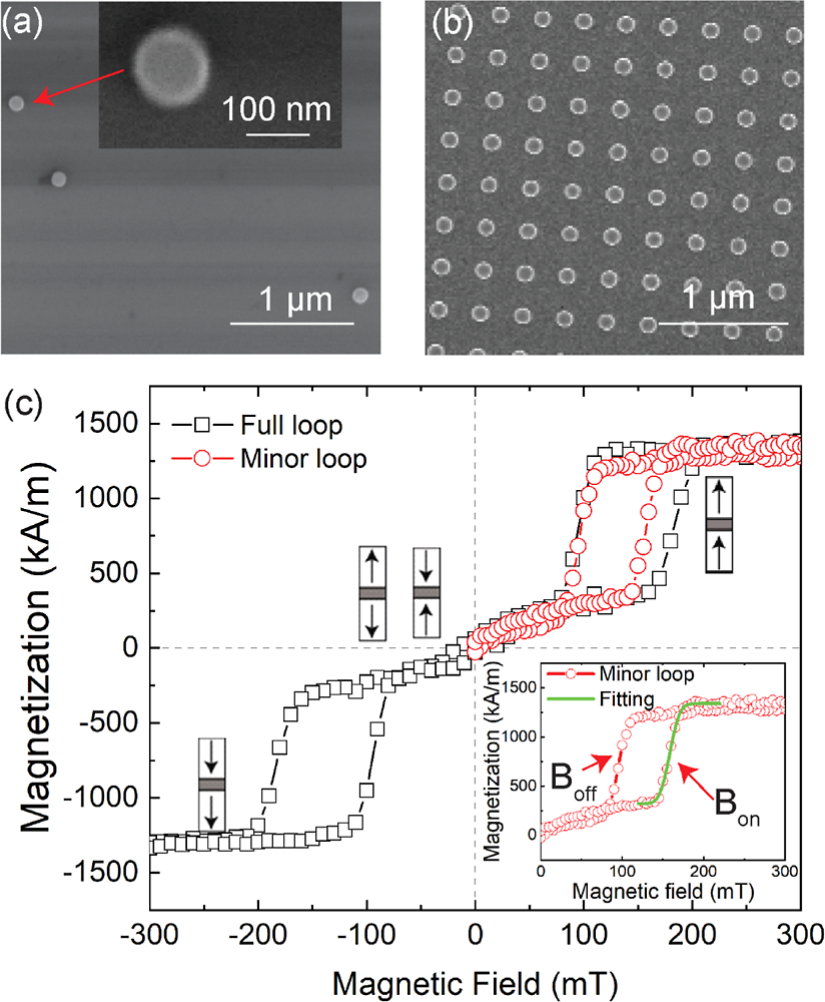
(a) SEM image of the released SAF NPs spin-coated on a silicon substrate. The released SAF NPs were used in PT MCD measurements. (b) SEM image of unreleased SAF NPs on the silicon wafer used for fabrication. The unreleased sample was used in SQUID measurements. The bright ring at the edge of each single particle in the SEM image is due to redeposition during fabrication and is an artifact due to an inclined electron exposure leading to enhanced secondary electron generation. (c) Hysteresis loops of the NPs measured with a SQUID at 400 K. The black squares represent the full hysteresis loop (i.e., major loop). The inset shows the fitting of the minor loop through an error function, from which we obtain the switching field and SFD (for details, see Section 4 in the Supporting Information). The red data set represents the minor loop. The black arrows indicate the direction of the magnetization of the top and bottom ferromagnetic layers constituting the SAF. The red arrows indicate on- (*B*_on_) and off- (*B*_off_) switching fields of the minor loop. The difference between the major and minor loops is due to the hysteretic effect of magnetization switching.

Magnetic nanoparticles (NPs) have shown promising applications in biomedicine.^1−6^ Among magnetic NPs, synthetic antiferromagnetic (SAF) systems with a large perpendicular anisotropy (PMA) are of interest in various nanoscale torque-transfer-related applications^7,8^ due to their large magnetic and shape anisotropy. The SAF structure is composed of two ferromagnetic layers which are antiferromagnetically coupled by a spacer layer through the Ruderman–Kittel–Kasuya–Yoshida (RKKY) interaction.^9−11^ In this case, the structure shows a 0 net magnetic moment at low applied magnetic fields. This specific feature of the SAF-PMA system prevents aggregation of NPs in solution. Under increasing magnetic field, the NPs switch from antiparallel (AP) to parallel (P), which we term the “on” field *B*_on_ switch, or vice-versa, which we term the “off switch” *B*_off_ (see Figure 1c). Note that by magnetization switching, we refer to magnetization switching under an applied magnetic field and not to optical switching of the magnetization.

Knowing the switching fields (*B*_on_ and *B*_off_), which consist of the RKKY coupling field *B*_rkky_ and a stochastic coercive field *B*_c_, needed to magnetically (de-)activate the NPs is a key requirement. Several reports^1,12^ have found that ensembles of PMA-SAF nanostructures are characterized by large switching field distributions (SFDs) reflecting the degree of particle-to-particle heterogeneity of their magnetic properties, whereas a well-defined *B*_on_ and *B*_off_ and narrow SFDs are preferred for applications. The broad SFDs are understood by considering the switching mechanism of ultrathin PMA nanostructures, assigned to stochastic thermally activated nucleation of a small magnetic domain, followed by fast domain wall propagation.^13−15^ These nucleation centers, which have variable density and broaden the SFD, are crystal defects in the nanostructure and fabrication-induced defects. Moreover, here we speculate that the dipolar field contribution to *B*_on_ is different and large, compared to its contribution to *B*_off_, leading to differently distributed *B*_on_ and *B*_off_ magnetic switching fields which we can conveniently probe at the single-particle level using PT MCD.

To understand the switching mechanism and broad SFDs of PMA nanostructures, measurements at the single-particle level are essential. However, most easy accessible characterization techniques which address the SFD are so far based on analyzing hysteresis loops measured on millions of particles simultaneously, so as to attain a sufficient signal-to-noise ratio.^16−19^ There are a few techniques, e.g., microsuperconducting quantum interference device (SQUID)^20,21^ and differential phase contrast and electron holography in transmission electron microscopy,^22,23^ which have been reported to measure SFDs at the nanometer scale. However, these techniques require demanding experimental conditions which are costly and/or complex in design. Recently, Spaeth et al.^24^ reported a simple optical technique, photothermal (PT) magnetic circular dichroism (CD) microscopy (PT MCD), which presents the sensitivity required to measure the magnetization of single magnetite nanoclusters with a diameter of about 400 nm. PT MCD is based on the polar magneto-optical Kerr effect (MOKE)^25^ and related to the imaginary part of the dielectric susceptibility. It measures the differential absorption of left- and right-circularly polarized light by a single-magnetic NP. Very recently, the sensitivity of PT MCD was improved sufficiently to measure the hysteresis loops of single 20 nm magnetite NPs.^26^

In contrast to previous reports on single synthetic magnetic NPs, in this report, we use PT MCD to study the switching behavior of 32 individual single top-down nanofabricated PMA-SAF NPs with a diameter of 122 nm and a thickness of 15 nm. We compare these signals with ensemble-based SQUID measurements to provide detailed insight into the variation of magnetic properties from NP to NP. Previously, the polar-MOKE effect on a single-particle level was reported on NPs with 2 μm diameter,^7^ which are more than 2 orders of magnitude larger in volume than our particles. Due to the high sensitivity of our PT MCD technique, we are able to measure the full magnetization-switching curve on each individual 122 nm particle using optical microscopy. We then compare the statistics of the switching events at the single-NP level, and observe a difference between AP → P (on-switching) and P → AP (off-switching). This difference is washed-out in the ensemble measurement. We speculatively attribute this difference to the presence of a dipole field contribution in the on-switching which is not present in the off-switching. The switching fields are also found to be broadly distributed among individual nanoplatelets indicating spatial heterogeneity. Moreover, a small difference between SQUID ensemble measurements and PT MCD is expected due to their respective time responses; here, the SQUID measurement was slow (∼hour) compared to PT MCD (∼minute) (see details in Section 4 in the Supporting Information). To address these differences further, we compare 15 successive loops on one and the same NP to study the temporal heterogeneity (stochasticity) of the switching process. We again observe a difference between the on- and off-switch, although it is less pronounced. Such a distinction between spatial and temporal heterogeneity can only be obtained from single-particle measurements because these two sources of heterogeneity are averaged out in ensemble measurements.

In this report, we show that single-particle PT MCD enables us to study magnetization properties of single magnetic nanoplatelets. We have found that magnetic switching fields are broadly distributed among individual nanoplatelets and also stochastic in nature, indicating spatial and temporal heterogeneity. In addition, the distribution of on- and off-switching fields is different, which we speculatively attribute to a dipolar contribution.

## Methods

The NPs used in the study consist of the following film stack: Ta(4)/Pt(2)/Co_80_B_20_(0.8)/Pt(0.4)/Ru(0.8)/Pt(0.4)/Co_80_B_20_(0.8)/Pt(2)/Ta(4) with thickness in nanometer indicated between parentheses (total thickness 15.2 nm). The stack was fabricated through magnetron sputter deposition on Si substrates and patterned via substrate conformal imprint lithography (SCIL) and a lift-off procedure into a liquid environment (see Supporting Information, Section 1 and ref (1)). The NPs dispersed in solution are termed “released”, and the NPs which are still attached to the substrate, i.e., before release, are termed “unreleased”.

SQUID magnetometry was used to obtain the hysteresis loop of unreleased NPs (Figure 1b), where an ensemble average of a total of ∼10^6^ SAF NPs was measured (see Supporting Information, Section 3). Note that the sample of unreleased NPs was used only for SQUID, not for the single-particle PT MCD measurements. During the measurement, a magnetic field was applied along the normal of the NPs at 400 K (the temperature of 400 K is chosen to match the temperature of single-particle PT MCD measurements). A minor loop (see the red data set in Figure 1c) was measured, where the samples were first saturated in a positive field. The magnetic field was then decreased to 0 and swept back to the positive saturation field. From the minor loop, the RKKY coupling field (*B*_rkky_) is defined as (B_on_ + *B*_off_)/2 and the coercivity (*B*_c_) is defined by (*B*_on_ – *B*_off_)/2. *B*_on_ and *B*_off_ are the switching fields from AP → P (on-switch) and from P → AP (off-switch), respectively, as depicted in Figure 1c.

In PT MCD measurements, a heating laser was used to illuminate the NPs (see details about the optical setup in Section 10 in the Supporting Information). The released NPs were dispersed on a glass substrate by spin-coating (see Figure 1a) and immersed in hexadecane, which was the contrast medium for PT imaging^27^ (see details about sample preparation in Section 9 in the Supporting Information). The difference in the absorption of the left- and right-circularly polarized light leads to a change of temperature and therefore to a change in the refractive index of the medium, which is detected by the probe beam. The measured CD signal is defined as σ_L_ – σ_R_, where σ_L_ and σ_R_ are the absorption cross sections of the NP for left- and right-circularly polarized light, respectively.^28^ The MCD signal is the CD signal due to the polar magneto-optical Kerr effect. Therefore, it reports on the particle’s absorption and its changes with magnetic field, and it depends only on the imaginary part of the optical susceptibility. The so-called *g*_CD_ factor is defined as the CD signal normalized by the PT signal (see Section 6 in the Supporting Information). Minor loops in both negative and positive fields are measured (more details are given in Section 11 in the Supporting Information). As the particles were heated with light, their temperature was estimated to be about 390 K (see further details in Section 7 in the Supporting Information).

## Results and Discussion

Scanning electron microscopy (SEM) images of released and unreleased NPs are shown in Figure 1a,b, respectively. The NPs have an average diameter of 122 ± 4 nm (see Figure S1). The unreleased NPs are used for the ensemble SQUID measurements and the released NPs for the PT MCD measurements. The SQUID measurements of major and minor loops in Figure 1c show the typical SAF behavior.^1,5,6,10^ At a low magnetic field, the total magnetization is 0 due to the antiferromagnetic coupling of the top and bottom CoB layers of nearly equal magnetic moments. Increasing the external field leads to an on-switch at *B*_on_ of one of the CoB layers, giving *B*_rkky_ = 127 mT and *B*_c_ = 32 mT, calculated from the minor loop. We observe a gradual switch in both the major and the minor loops. This gradual switch reflects the SFD of ∼10^6^ single NPs. By fitting the (minor) hysteresis loop with an error function (see Figure 1c), we extract the SFD of the ensemble (more details are given in Section 4 in the Supporting Information). The center value of the fits represents the switching fields *B*_on_ and *B*_off_, (158 ± 10) mT and (95 ± 10) mT, respectively (see Table 1). Note that the difference between the major and minor loops, as shown in Figure 1c, is due to hysteresis.

**Table 1:** Switching Fields (*B*_on_ and *B*_off_) and Their Distribution, *B*_rkky_ and *B*_c_, Measured by SQUID and PT MCD

	*B*_on_ (mT)	SFD of *B*_on_ (mT)	*B*_off_ (mT)	SFD of *B*_off_ (mT)	*B*_rkyy_	*B*_c_
SQUID at 400 K	158	10	95	10	127	32
PT MCD at ∼390 K	114	25	61	5	88	27

**Figure 2:**
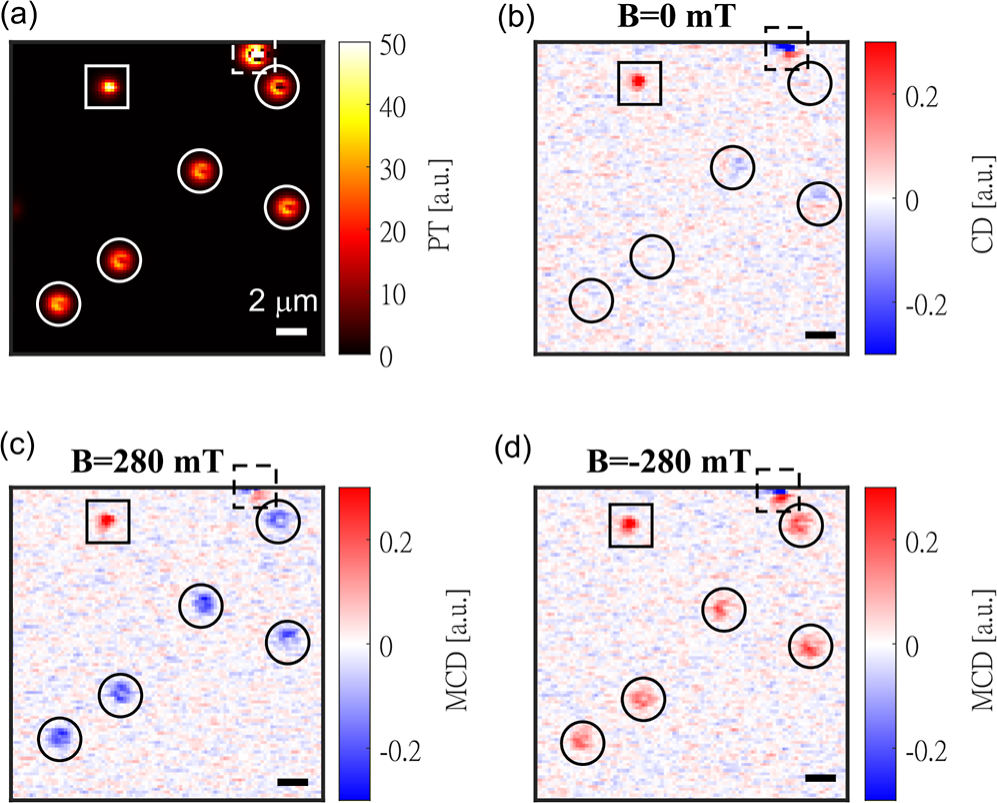
(a) PT imaging of magnetic NPs. Single NPs are identified by the homogeneous magnitude of their PT signals (solid circles). An aggregate, marked with a dashed square, has much stronger PT signal. (b) CD imaging of magnetic NPs in the absence of an applied magnetic field (*B* = 0). Single NPs show very weak CD signals at *B* = 0 mT. MCD imaging of magnetic NPs at (c) *B* = 280 mT and (d) *B* = −280 mT. The MCD signal flips sign upon inversion of the magnetic field’s orientation. The particle marked with a solid square shows no flip of MCD signal with flip of magnetic field orientation. This particle is probably not a SAF particle. The scale bars are 2 μm.

Figure 2a shows a PT image of single released magnetic NPs spin-coated on a glass substrate. Single magnetic NPs are identified by the magnitude of their PT signals, which falls in a very narrow range (see the histogram of PT signals in Figure S3). These single NPs are marked with solid circles in Figure 2. Their point-spread-functions (PSFs) are very similar for all NPs measured, as expected from their narrow size distribution. We attribute the complex shape of the PSF of a single platelet to interference between probe waves scattered by the thermal lens and by the particle itself.^27^ We found a few aggregates of NPs, which we identified by their stronger PT signals, as indicated in Figure 2a with a dashed square. Such aggregates were not considered in the analysis of our results. MCD images of the same NPs in applied magnetic fields of *B* = 0 mT, *B* = 280 ± 6 mT, and *B* = −280 ± 6 mT are shown in Figure 2b–d, respectively. Single NPs show weak CD signals in 0 applied magnetic field (see Figure 2b), indicating that they are structurally symmetric and present 0 net magnetization. Under a high magnetic field where NPs are saturated (*B* = 280 ± 6 mT and *B* = −280 ± 6 mT), a strong MCD signal is observed, as shown in Figure 2c,d. The MCD signal of the NP changes in sign upon inversion of the magnetic field, which distinguishes it from CD originating from shape and/or composition defects. The MCD sign depends on the wavelength of the light. At 532 nm, it is negative for a positive applied field, in our sign convention (see Methods). We also observe few particles (e.g., marked by a solid square in Figure 2) which show strong CD signals in the absence of an applied magnetic field but do not show any reversal of the CD signal with magnetic field. These particles were probably not single SAF NPs and were not considered in our later analysis. Identification and distinguishing of single SAF NPs from aggregates and other types of magnetic NPs are advantages of the single-particle technique. The variation of the MCD signal with the applied field opens up the possibility to measure hysteresis loops at the single-particle level.

**Figure 3:**
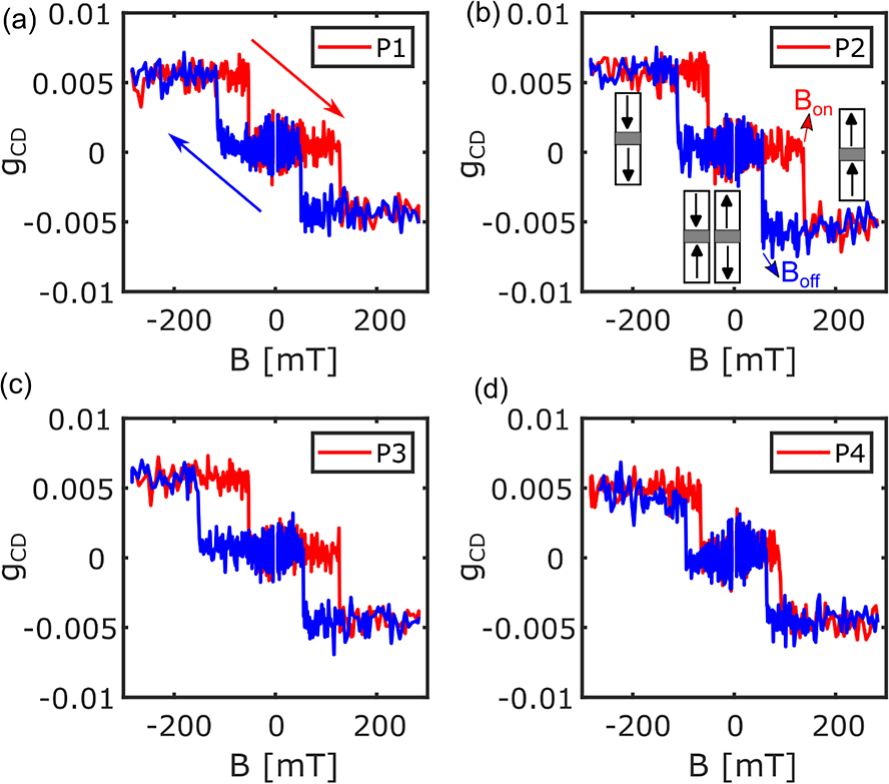
(a–d) Full magnetization curves of four single magnetic NPs, labeled as P1, P2, P3, and P4. Schematic of spin-flip is shown in (b) with on- and off-switching fields labeled with *B*_on_ and *B*_off_.

We now consider the hysteresis loops of single magnetic NPs, as shown in Figure 3a–d. A total of 32 single magnetic NPs were measured (see Figures S6 and S7). The first thing we observe is that all 32 single NPs show characteristic PMA-SAF behavior, i.e., the AP to P switch at *B*_on_ and from P to AP at *B*_off_ and 0 MCD around 0 applied field. In contrast to the ensemble hysteresis loop, we now find that all switching events observed are sharp as observed in the continuous film samples (see Supporting Information, Section 5 and Figure S2), in agreement with the proposed switching mechanism of a single NP, which starts with domain nucleation and is followed by propagation of the domain wall.^12^ This is a new insight compared to the SQUID measurements, where the broad SFD reflected the distribution of the ensemble (i.e., particle-to-particle SFD) but provided no clear indication as to the sharpness of each individual switching event (i.e., of the single-particle SFD). Such a distinct information can only be obtained from single-particle measurements. The apparently higher noise observed at low fields in the hysteresis loop is a measurement artifact due to the denser sampling at low fields than at high fields (for details, see Supporting Information, Sections 13–14 and Figures S8 and S9).

The histograms taken from 32 NP measurements of the PT and *g*_CD_ factors at saturation are presented in Figure S3a,b. They show comparatively narrow distributions, consistent with the high monodispersity of NPs (see Figures 1 and S1). This means that all NPs have similar *g*_CD_ factors of about 5 × 10^–3^ at saturation (see Figure S3b). The MCD signal arises mainly from the magnetic layers of the NP, whereas the PT signal arises from the absorption of both the magnetic and nonmagnetic layers (see a schematic of layers in Figure S11). Simulations discussed in the Supporting Information (Section 17) suggest that only ∼8.7% of the total light is absorbed in the two CoB layers. Therefore, normalization of the *g*_CD_ factor on the total CoB absorption would yield a value of (5 × 10^–3^)/0.087 i.e., 5.7 × 10^–2^. In a previous report,^24^ we have found that the saturation *g*_CD_ factor of magnetite NPs was ∼1 × 10^–2^. This difference can be related to the difference in the saturation magnetization (*M*_s_); however, due to the complexity of magneto–optic interactions,^29^ the absolute mapping of the *g*_CD_ factor to *M*_s_ is beyond the scope of this study.

**Figure 4:**
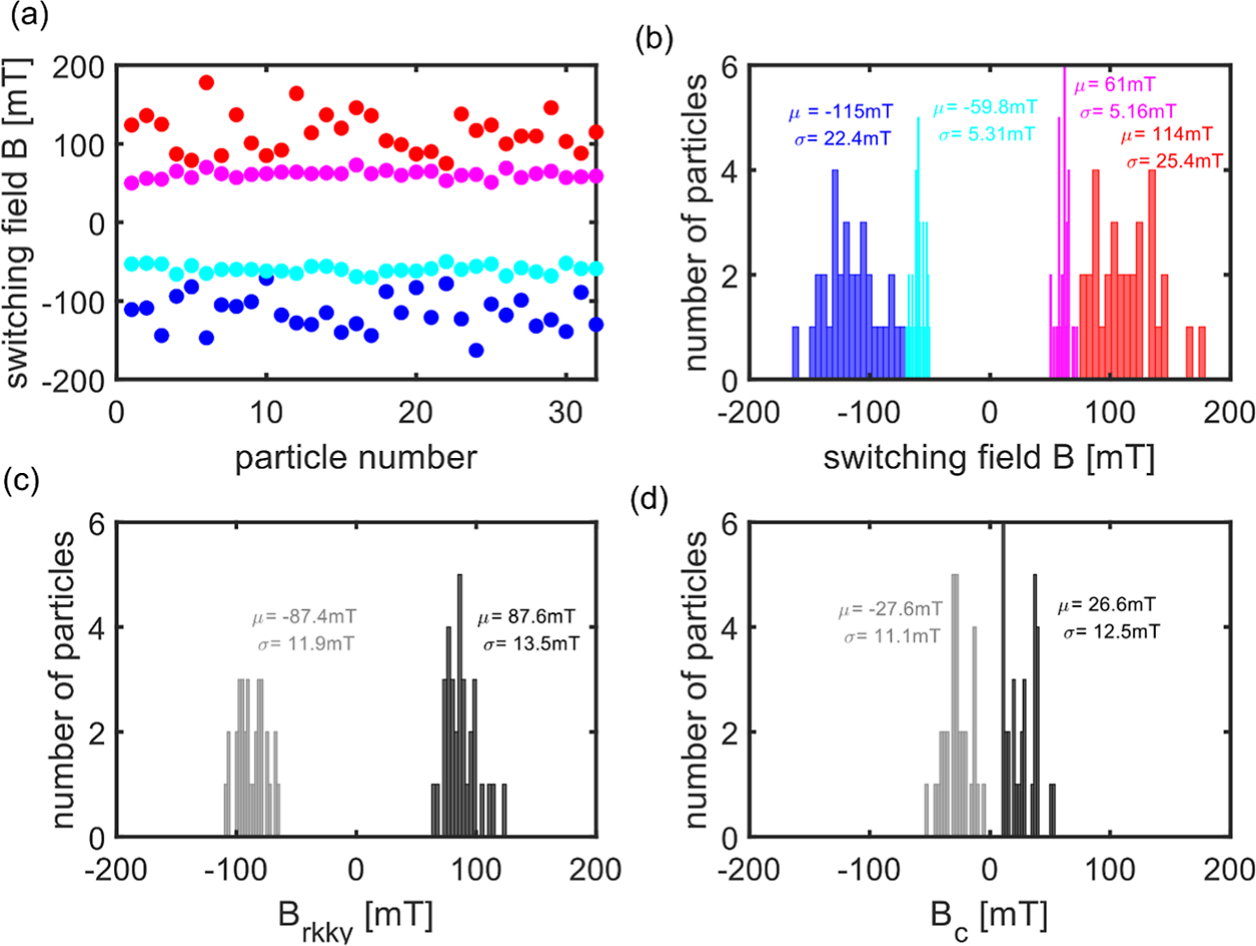
(a) Low positive (magenta) and negative (cyan) switching fields and high positive (red) and negative (blue) switching fields for each particle. (b) Histograms of all the switching fields. (c) Histograms of positive (black) and negative (gray) coupling fields *B*_rkky_ i.e., (*B*_on_ + *B*_off_)/2 where *B*_on_ and *B*_off_ are high and low switching fields, respectively. (d) Histogram of positive (black) and negative (gray) coercive fields *B*_c_, i.e., (*B*_on_ – *B*_off_)/2. Mean values (μ) and standard deviations (σ) of the histograms are shown in the inset.

We now compare the statistics of the switching behavior of the 32 single NPs measured by PT MCD with the ensemble SQUID measurement. The switching fields *B*_off_ and *B*_on_ of 32 single NPs and their histograms are shown in Figure 4a,b. The mean values and distribution of the histograms of *B*_on_ and *B*_off_ are summarized in Table 1. The histograms of *B*_rkyy_ and *B*_c_ are shown in Figure 4c,d, with mean values in Table 1.

In agreement with our SQUID measurements, PT MCD measurements of single NPs show that these switch at different fields (see Figure 4a,b), giving rise to a broader SFD for *B*_on_ (25 mT) as compared to the SQUID (10 mT). The SQUID measurements were performed on a 4 × 4 mm^2^ piece of wafer, whereas the PT MCD measurements were done on released NPs from a full 2 in. wafer. We thus expect more inhomogeneity and a broader SFD for the released NPs because they originated from the whole wafer and sampled the full inhomogeneity of the deposition process. We attribute the difference of the absolute values of *B*_off_, *B*_on_, and *B*_rkky_ between PT MCD and SQUID to the error in the estimated temperature of the platelets in PT MCD measurements and/or to small differences in the calibration of the magnetic field (estimations of temperature in PT MCD and the effect of temperature on switching fields are given in Supporting Information, Sections 7 and 8 and shown in Figures S4 and S5). In addition, the difference may arise due to the change in strain in the released particles by the lift-off process compared to the unreleased particles. The measured *B*_c_ in PT MCD (27 mT) and SQUID (32 mT) match well as *B*_c_ is a relative measurement of the two switching fields.

Interestingly, the SFD is much broader for *B*_on_ (26 mT) than for *B*_off_ (5 mT) in the single-particle PT MCD measurements, whereas they are similar (both 10 mT) for the SQUID measurements. This can be speculatively explained by the dipole fields in the AP configuration, as schematically shown in Figure S13, and by the role of residual nucleation embryos in the reversal mechanism of on- and off-switching.^30−32^ When NPs switch from AP to P state (*B*_on_), the dipole fields of the two CoB layers repel each other, leading to a canting of the magnetization at the edge of NPs, which, in turn, assists domain nucleation. In the P to AP switching (*B*_off_), the dipole fields are aligned and do not contribute to the nucleation process. In addition, irreversible nucleation embryos left over from former field cycles may further contribute to the SFD.^32^ In contrast to PT MCD, the reduced *B*_on_ was not observed in SQUID, possibly because the effect is averaged out on a large number of NPs. Note that the dipolar interaction between NPs (not the interparticle dipole field) in both the PT MCD and SQUID measurements can be neglected since the dipolar field of neighboring NPs is very small (see Supporting Information, Section 18 and Figure S12). This difference needs further investigation. These findings, however, illustrate the power of single-particle measurements to reveal details of the single-particle switching properties, compared to ensemble studies.

**Figure 5:**
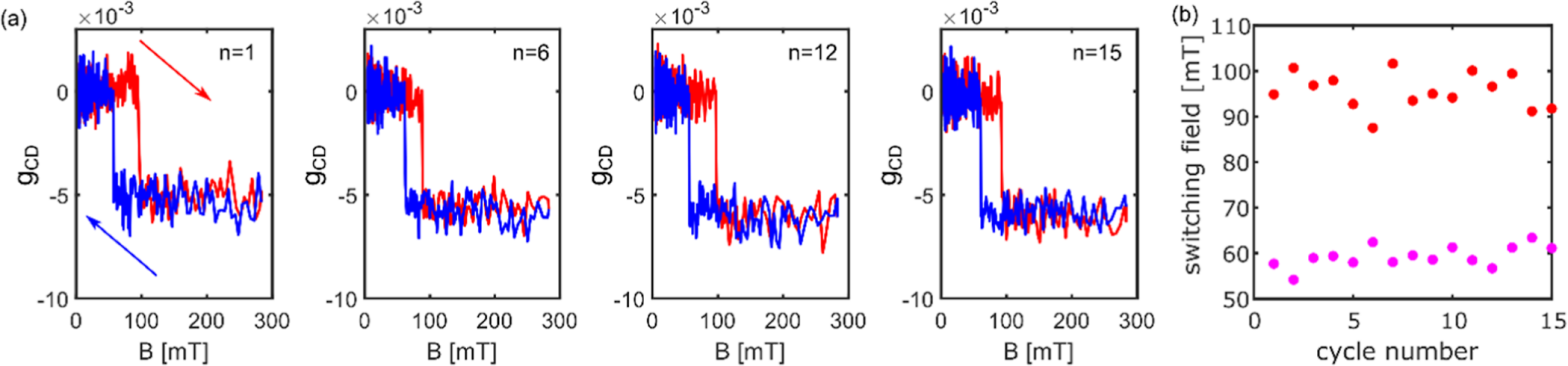
(a) Time-dependent minor loops of magnetization curves measured successively 15 times on the same single NP. Here, only the 1st, 6th, 12th, and 15th cycles are shown. (b) On- (red) and off- (magenta) switching fields measured for each cycle.

In addition to the SFD measured on an ensemble of single particles, we also measured the same single particle repeatedly for 15 times. The minor loops of several cycles are shown in Figure 5a. Both *B*_off_ and *B*_on_ fluctuate from cycle to cycle, as shown in Figure 5b. We assign the temporal fluctuation of the switching field to the thermally activated stochastic domain nucleation process (the SFD is shown in Figure S10).^11^ The mean values (standard deviations) are ∼59 ± 2 mT and about 96 ± 4 mT for *B*_off_ and *B*_on_, respectively. The cycle-dependent fluctuations of *B*_on_ are larger than those of *B*_off_. The *B*_off_ fluctuations are similar to those found on the 32 different particles, as shown in Figure 4. However, the *B*_on_ fluctuations for the 32 NPs are much larger than those for the single NP, implying that more disorder is found in the small ensemble of 32 NPs, which includes both spatial and temporal disorder. Thus, our PT MCD method enables us to distinguish spatial and temporal heterogeneity in the switching behavior of magnetic NPs.

## Conclusions

In summary, we have shown that single-particle PT MCD is a very powerful technique to study the magnetization switching of single magnetic PMA-SAF NPs. The measured SAF properties of NPs and the narrow distribution of the PT MCD of NPs indicate that the PT MCD is a powerful probe of the magnetic behavior of individual NPs. Moreover, compared to SQUID, the spatial and temporal heterogeneity of the magnetic properties, especially the switching fields at the single-particle level, can be extracted from PT MCD. The SFD generated by averaging the switching field of many NPs via the PT method is found to be broad. In addition, the minor loops successively measured on the same NP vary moderately from cycle to cycle, confirming that the reversal process is indeed a thermally activated stochastic process. We observed a difference in the magnetization switching from AP → P vs P → AP in the PT measurements, which was absent in the SQUID measurements. We speculatively attribute this difference to the dipole-field which assists reversal for AP → P (on-switching) but is absent for P → AP (off-switch). Such details are washed out in ensemble measurements.

## Data Availability

The data that support the findings of this study are available upon reasonable request from the authors.

## Supporting Information Available

Nanofabrication of SAF-PMA and dispersion in a liquid; size distribution of NPs; SQUID measurement; error function fit to the magnetization curve obtained from SQUID; hysteresis loop of the continuous SAF film; histogram of PT signals and g_CD_-factors; calculation of temperature in PT MCD using a calibration method; temperature-dependent switching fields; sample preparation for PT MCD measurement; optical setup; measurement sequence of applied magnetic fields in PT MCD measurement; full magnetization curves of 32 single magnetic NPs; magnetization as a function of the permanent magnet’s position; MCD time traces at *B* = 0 and *B* = 280 mT; histogram of low and high switching fields from time-dependent magnetization curve; simulation for calculating absorption of magnetic layers; effect of dipolar field on the unreleased SAF NPs in SQUID measurement; and schematic representation of the spin reversal mechanism (PDF)
