# Supplementary material for: Optical Monitoring of the Magnetization Switching of Single Synthetic-Antiferromagnetic Nanoplatelets with Perpendicular Magnetic Anisotropy
Source: ACS Photonics. 2023 Apr 28;10(5):1512–8. doi: 10.1021/acsphotonics.3c00123 (PMC10197163; doi:10.1021/acsphotonics.3c00123)
Supplement: Supplementary file 1 — ph3c00123_si_001.pdf [file ph3c00123_si_001.pdf]

Supporting Information for

Optical Monitoring of the Magnetization Switching of Single Synthetic-Antiferromagnetic Nanoplatelets with Perpendicular Magnetic Anisotropy

S. Adhikari[3,†], J. Li[1, †], Y. Wang[3,4, †], L. Ruijs[1], J. Liu[4], B. Koopmans[1], M. Orrit[3,]*

and R. Lavrijsen[1,2,]*

† *Contributed equally*

* *r.lavrijsen@tue.nl*

* *Orrit@physics.leidenuniv.nl*

1. Department of Applied Physics, Eindhoven University of Technology, P.O. Box 513, 5600 MB Eindhoven, Netherlands

2. Institute for Complex Molecular Systems, Eindhoven University of Technology, P.O. Box 513, 5600 MB Eindhoven, The Netherlands

3. LION, Huygens-Kamerlingh Onnes Laboratory, 2300 RA Leiden, Netherlands

4. School of Mechatronics Engineering, Harbin Institute of Technology; Harbin 150001, P. R. China

Number of pages: 23

Number of figures: 13

1. Nanofabrication of SAF-PMA and dispersion in a liquid

A substrate conformal imprint process was used to fabricate the SAF-PMA nanoplatelets (NPs) which were studied in the paper. After depositing the sacrificial Cu layer and the SAF thin film stack on a 2-inch silicon wafer, the disc shape was created by the imprint process. Several etching processes e.g. reactive ion etching, ion beam milling and buffered hydrogen fluoride etching were used to transfer the disc shape into the metal layer. Then the Cu layer was dissolved by $CuSO_4$-ammonia solution to release the NPs. After release into the liquid environment, the NPs were cleaned with deionized (DI) water three times. Then the NPs were redistributed in the DI water with sonication. For the SEM measurement, the released NPs were prepared by spin coating the solution which contained the NPs on a silicon wafer at 500 rpm. For the unreleased sample, a piece of wafer of the sample before release was used.

2. Size distribution of nanoplatelets

The size distribution of nanoplatelets is calculated based on the TEM image of 146 released nanoplatelets. To prepare the TEM sample, the nanoplatelets were dispersed in DI water and drop cast on a TEM copper grid. One example of the TEM image of the released NPs is shown in Fig S1(a). Only the NPs which were fully aligned on the substrate were taken into consideration. ImageJ was used to analyze the diameter of the NPs and a histogram of the diameters of the nanoplatelets is shown in Fig S1(b). A normal distribution is used to fit the diameter and we obtain a size distribution of 122 ± 4 nm.

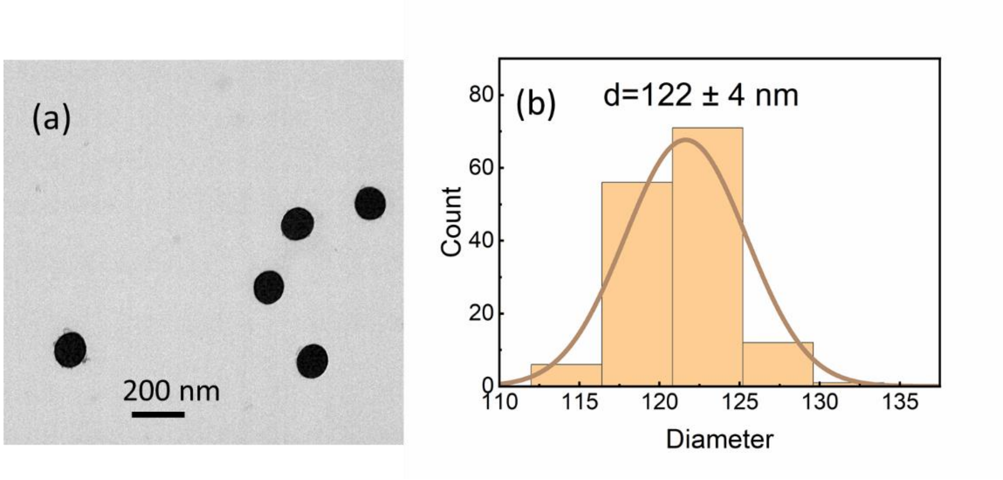

Figure S1: (a) TEM image of released SAF nanoplatelets. (b) Histogram of the diameters of the nanoplatelets. A normal distribution is used to fit the histogram.

3. SQUID measurement

The SQUID measurement of the SAF nanoplatelets was conducted on the unreleased NPs, which were still on the silicon wafer, instead of the released NPs. A 4×4 mm^2 wafer piece of the unreleased sample was used in the measurement. Considering the filling rate of NPs shown in Fig. 1b, in total ~ 10^6 SAF nanoplatelets were measured. A magnetic field was applied perpendicular to the sample surface. The reason for using unreleased nanoplatelets is to prevent the misalignment of the dried-in nanoplatelets relative to the applied field and the possibility of nanoplatelets piling on top of each other, which can cause more slanted switching fields due to particle-particle interaction.[1] Previous study also denotes that after release the typical SAF properties do not degrade,[1] indicating that the properties of unreleased nanoplatelets measured by SQUID can be compared with the properties of released nanoplatelets measured by PT MCD.

4. Error function fit to the magnetization curve obtained from SQUID

In the SQUID measurement, the ensemble average switching behavior of more than 10^6 particles was measured. Since the switching process is a stochastic process, an error function ($y = A \cdot \mathrm{erf}\left(\frac{x - B_{sw}}{\sqrt{2}\, SD}\right) + B$, where A and B are constants and B_{sw} is the average switching field and SD is the standard deviation) was used to fit for part of the hysteresis loop as shown in the insert of Fig 1c. The standard deviation of the fit represents the switching field distribution (SFDs) of the ensemble, dominated by NP to NP variation. Moreover, as SQUID is a quasi-static measurement process, i.e. field is set, a measurement is performed, etc, the time interval between two points is on the order of 20 seconds. In the PT MCD measurement a continuous measurement protocol is used, i.e. the field changes continuously and points are measured while sweeping (the magnetic field is varied by moving the external magnet away from, or towards, the sample with a speed of 0.5 mm/s and the magnetic field is calculated from a calibration curve of field-distance measured with a Gauss Meter). Hence, the quasi-static SQUID does not probe the SFD of the individual NP (more time to perform a switch i.e. Arrhenius type of process see Eq 1.), but probes the distribution in the ensemble. In contrast the PT MCD protocol, where a continuous measurement is performed, which for an Arrhenius process leads to a larger spread as the measurement time is shorter, indeed probes the switching process of individual NPs and reflects the single-particle SFD.

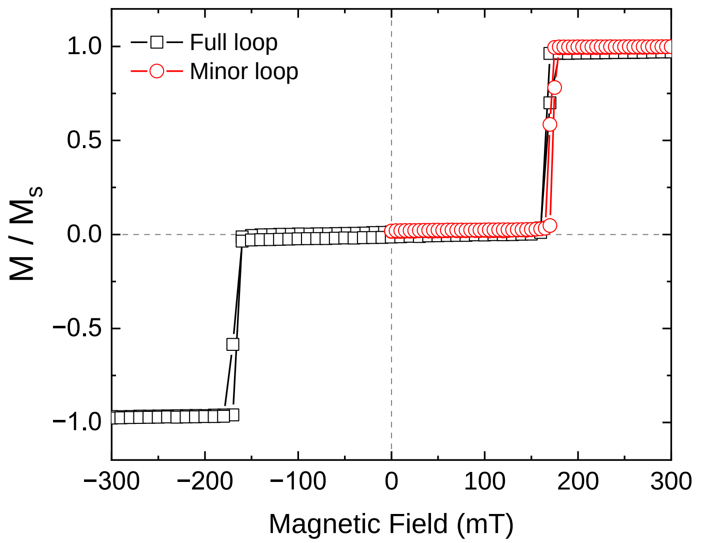

<u>Figure S2:</u> Hysteresis loop of the as-deposited continuous film SAF stack at 400 K.

<u>5. Hysteresis loop of a continuous SAF film</u>

The hysteresis loop of the as-deposited continuous film of the SAF stack before patterning is measured by SQUID at 400 K as shown in Fig. S2. SAF properties are observed with the B_{rkyy} of 171 mT. Sharp switches with the SFD of 2 mT from AP to P state and P to AP state are observed compared to the patterned SAF NPs which exhibit a broad SFD of 10 mT (see Fig. 1c). The B_c of patterned NPs is 22 mT while the B_c of the continuous field is 2 mT. The differences between the continuous film and the patterned NP are expected and can be attributed to the reversal mechanism. Since the switch starts with a reversal of nucleation followed by fast domain wall propagation, sharp switches are expected as we observed on the continuous film and on the individual NPs. However, the SQUID measurement of the patterned NPs contains many NPs thus the broader SFD can be observed. With reduced area, less nucleations can be found on the

patterned sample compared to the continuous film leading to a larger B_c of the patterned SAF NPs.

6. Histogram of PT signals and g_{CD} factors

Histograms of photothermal (PT) signals and g_{CD} factors of 32 single magnetic nanoplatelets at a saturated magnetic field are shown in Fig. S3. The g_{CD} factor is defined as

$$g_{CD} = \frac{CD}{PT} = 2\,\frac{\sigma_L - \sigma_R}{\sigma_L + \sigma_R}.$$

Where, *g_{CD} factor* is the normalization of CD signal by PT signal. σ_L and σ_R is the absorption cross sections of left- and right-circularly polarized light, respectively.

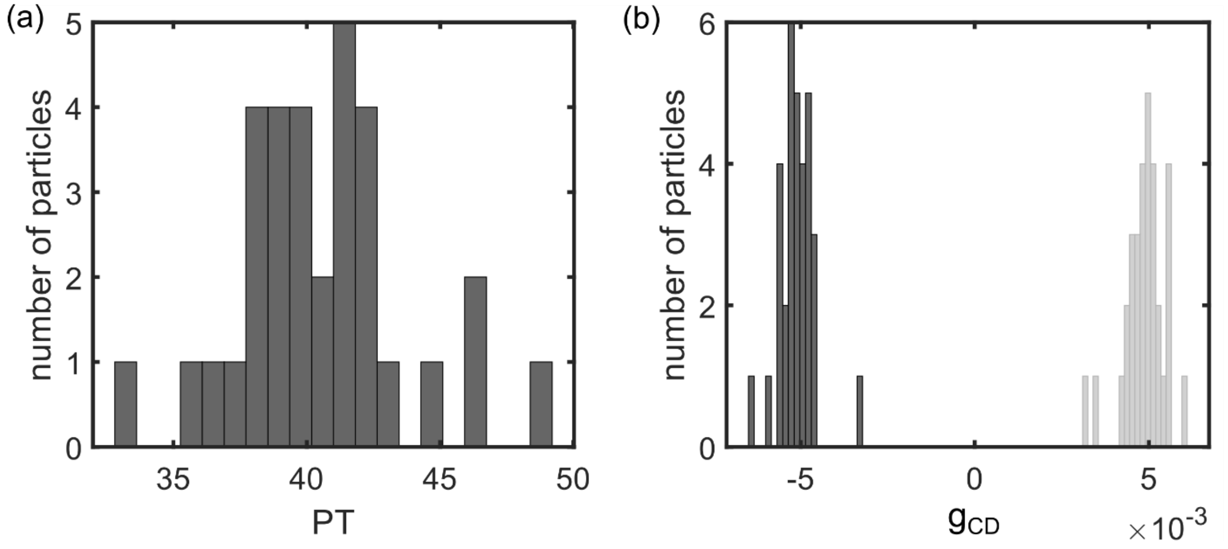

Figure S3: (a) Histogram of photothermal signals of 32 single magnetic nanoplatelets. (b) Histograms of g_{CD} factors at saturation magnetic fields of 280 mT (black) and -280 mT (gray).

7. Calculation of temperature in PT MCD using a calibration method

The temperature calibration was done by heating the nanoplatelets until nanobubble formation was observed in the liquid (Hexane) surrounding the NPs. The hexane has a lower boiling point

(341.6K) than hexadecane (560K) and was used as the contrast medium for this calibration. We performed the calibration with the following assumptions:

1. The nanoplatelets are assimilated to spheres.
2. The absorption cross section for the heating beam and for the probe beam are approximately equal.
3. The thermal conductivity and refractive index of hexane and hexadecane are nearly identical.

Then, we increase the heating power slowly until the PT signal suddenly increases significantly, which indicates nanobubble formation. Considering surface tension and size of the NP, the critical temperature of NP from calculation is 377 K. The critical power of heating and probe beam used in experiments are 7.5 mW and 1 mW, respectively. Because of the linear relationship between heating power and the temperature of nanoplatelet, the estimated temperature of nanoplatelet in hexadecane is 390.28 K with a heating power of 12 mW and probe power of 1 mW.

8. Temperature dependent switching fields

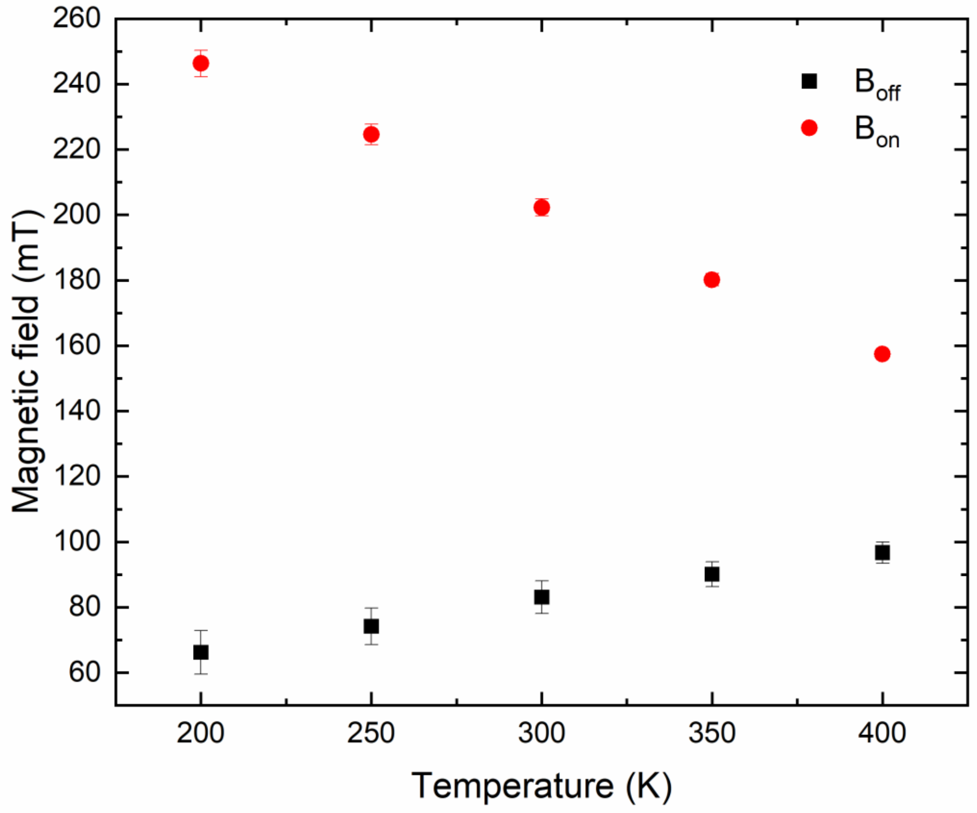

Figure S4: Temperature dependent switching fields (B_{on} and B_{off}) measured by SQUID.

Hysteresis loops were obtained by SQUID at temperatures ranging from 200 K to 400 K with an out-of-plane configuration on an ensemble of approximately 10^6 particles in a periodic configuration on a 4×4 mm^2 wafer piece. The measurements were performed several times over multiple days. Next to that, the temperature was first increased and then decreased for several times by which it was concluded that there was no irreversible effect observed in the magnetic properties for temperatures ranging up to 400 K. The switching fields of the minor loop as a function of the temperature are recorded in Fig. S4, the corresponding coercivity (B_c) and RKKY coupling field (B_{rkky}) are shown in Fig S5a and Fig S5b. The error bars of the B_c and B_{rkky} come

from the standard deviation of the values obtained from different measurements. We observe a decreasing coupling field with an increase in temperature as shown in Fig S5b, due to the switching fields moving closer together as can be seen in Fig. S4.

Besides that, the B_c is also decreased with an increase in temperature, which is the result of the thermally assisted switching behavior. The decrease in B_c can be fitted with the Sharrock formalism,

$$B_c = \frac{2K}{M_s}\left(1 - \left(\frac{k_B T}{KV}\ln\left(\frac{f_0 t}{\ln 2}\right)\right)^{\frac{1}{2}}\right) \qquad \text{Eq.1}$$

Here B_c is the coercive field [mT], K is the anisotropy constant [J/m^3], M_s is the saturation magnetization [A/m], k_B is the Boltzmann constant [J/K], T is the temperature [K], V is the nucleation volume [m^3], f_0 is the attempt frequency [1/s] and t is the measurement time [s]. From this equation it follows that for finite temperatures the coercive field is reduced by the factor in brackets with respect to the anisotropy field $\frac{2K}{M_s}$. For the fitting, f_0, t and k_B are fixed, where f_0 is set to 10^{10} Hz (typical for ferromagnets as CoB) and t is set to 20 s (~measurement time between points in the SQUID). Furthermore, it is assumed that both K and M_S have no temperature dependence which is a rather crude assumption for ultrathin CoB layers with a Curie temperature of ~550 K. The fitting parameters are then $\frac{2K}{M_s}$ and KV. The fitting gives that $\frac{2K}{M_s}$

equals 240 mT and KV equals $1.9 \times 10^{-19}\,J$. According to the fitting, the B_c at 390 K is 33 mT, which agrees nicely with the value obtained in the PT MCD.

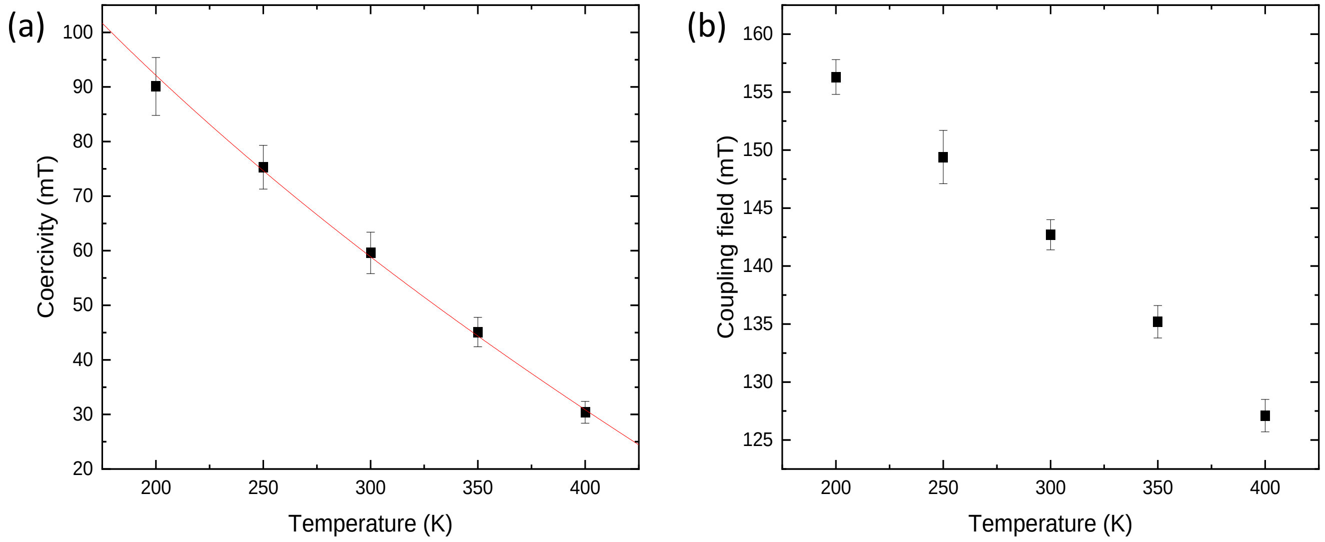

Figure S5: (a) Average temperature-dependent coercivity of an ensemble of ~10^6 platelets as obtained from the minor loops measured using SQUID. The data points are fitted using the Sharrock formalism as mentioned in the text. (b) Average temperature-dependent coupling field of an ensemble of ~10^6 platelets as obtained from the minor loops measured using SQUID.

9. Sample preparation for PT MCD measurements

The nanoplatelet solution was spin-coated on a UV-plasma-cleaned glass coverslip (thickness about 170 µm) to disperse the nanoplatelets homogeneously on the glass surface. The glass coverslip was sandwiched with a microscope glass slide with a cavity (glass slide's thickness of about 1.4 mm) which contained a cavity to hold the liquid (hexadecane, Sigma-Aldrich, MGPB20) used for the photothermal measurement.

10. Optical setup:

The details of the optical setup are described in our recent publication.[2] Here we describe the setup briefly. The heating laser with wavelength of 532 nm was passed through two polarization modulators, an electro-optical modulator (EOM) and a photo-elastic modulator (PEM) to modulate the laser's polarization at frequencies 33.5 kHz and 50 kHz, respectively. The circular dichroism (CD) signal was detected at the sum frequency. The heating laser was focused in the back-focal plane of the immersion-oil objective (NA = 1.45) to illuminate the sample in a wide-field area of about 3 μm diameter. The circularly polarized continuous-wave probe beam at wavelength 780 nm was focused onto the sample using the same microscope objective. The scattered probe beam was filtered from the heating beam using a band-pass filter (BP780) before focusing on an analog photodiode using a lens. A sensitive lock-in amplifier was used to obtain the CD signal at the sum frequency of two modulators. To vary the magnetic field, a long permanent cylindrical NdFeB magnet made from a set of small cylindrical magnets of diameter of 6 mm, was placed perpendicular to the sample plane and its position relative to the sample was varied to vary the applied magnetic field. The magnetic field was calibrated with a Hall probe (Hirst Magnetics, Model: GM07). The field was measured without the objective (the presence of the steel ring in the microscope objective may modify the magnetic field in the sample, however the influence of the steel ring was not considered due to the position of the ring several millimeter away from the sample). To invert the direction of the magnetic field, the magnet was reversely mounted. Note that PT MCD is most suitable for nanoparticle measurements and not very suitable for a thick film measurement. For a film measurement, it is quite complex to quantitatively understand and requires an additional theoretical modeling.

11. Measurement sequence of applied magnetic fields in PT MCD measurements

Upon measuring the hysteresis loop of NPs through PT MCD, g_{CD} factors under different applied fields, defined as CD signal normalized by PT signal, are recorded. A high positive magnetic field was applied to saturate the sample, then the applied magnetic field was varied from zero to high magnetic field, then returned to zero and then the same was done for the opposite orientation of the magnetic field. Thus, the hysteresis loops in both positive and negative magnetic field directions resemble the minor loop of the hysteresis curve measured by SQUID. Note that, in this study, we focus on the minor loops of the system, i.e., single-polarity sweeps of the field which will assure that only one of the two layers of the SAF system switches which allows us to compare the same switching event consistently.

12. Full magnetization curves of 32 single magnetic nanoplatelets:

The full magnetization curves of a total of 32 single magnetic nanoplatelets are shown in Fig. S6 and Fig. S7. All the platelets show sharp transitions from one of the anti-ferromagnetic configurations (on switch) to the ferromagnetic one and always reverted to an anti-ferromagnetic configuration upon field decrease (off switch).

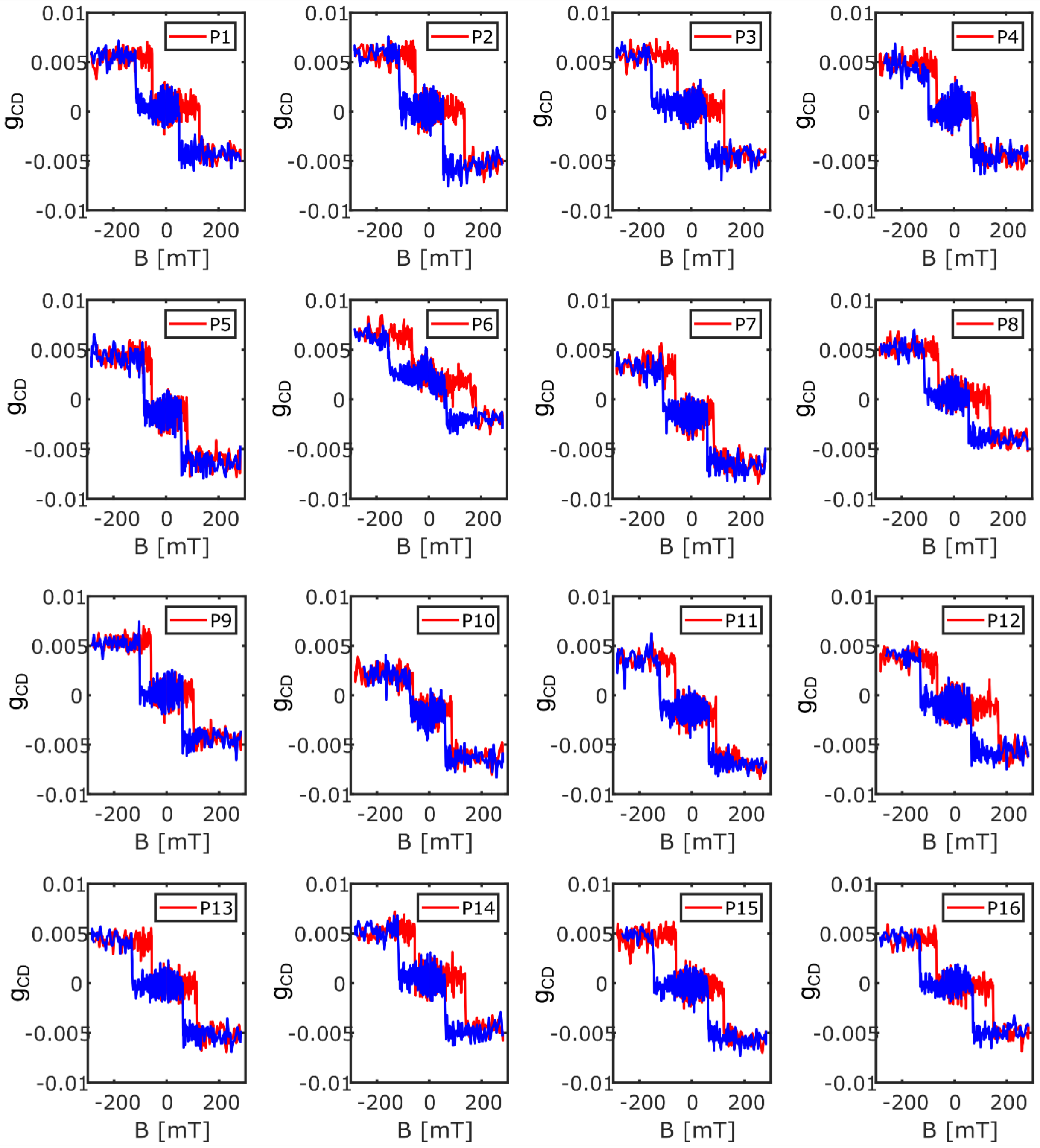

Figure. S6: Full magnetization curves of 16 single magnetic nanoplatelets. Magnetization curves of another 16 single nanoplatelets are shown in Figure S7. Particles numbers are mentioned in the inset.

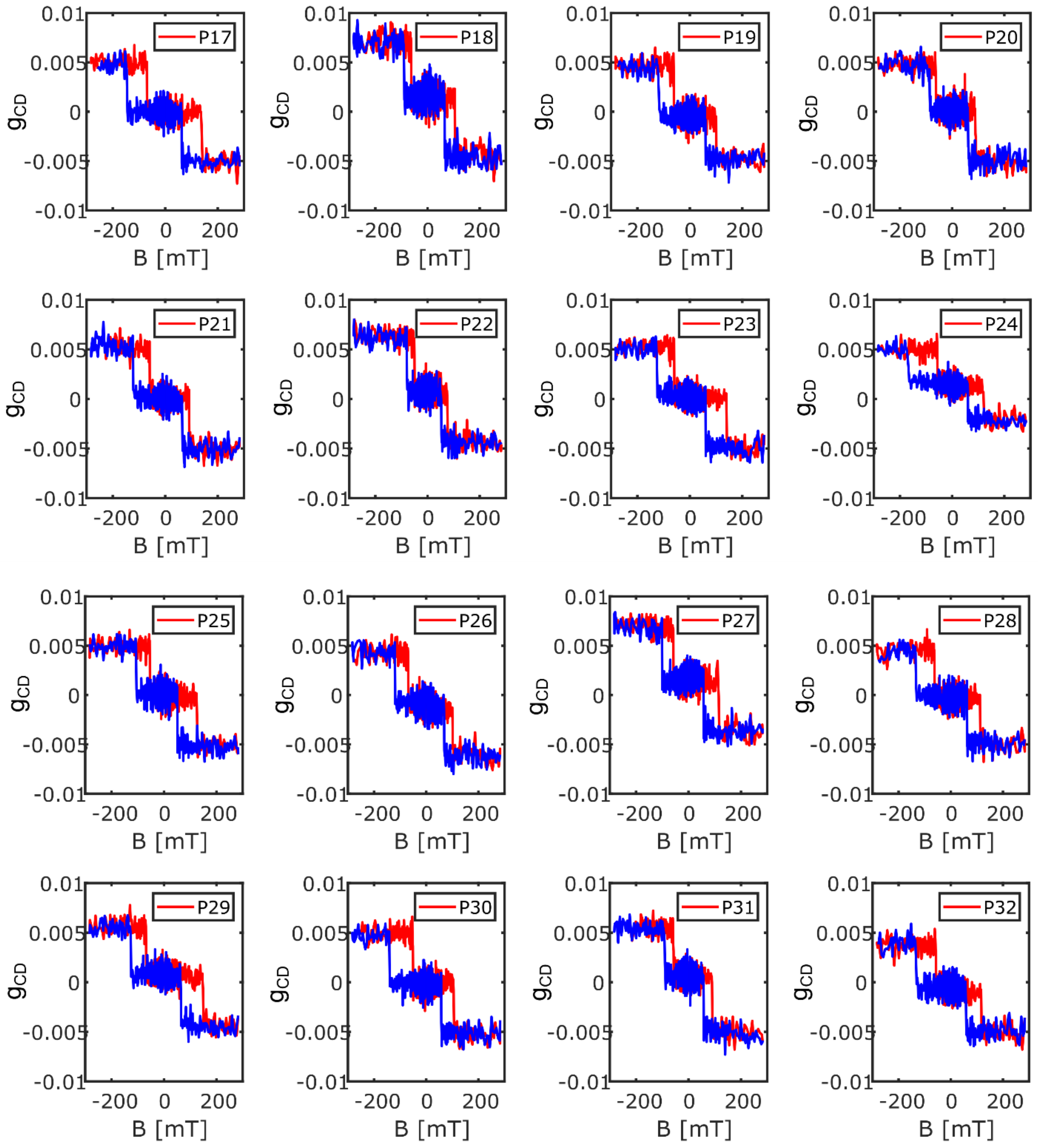

Figure. S7: Full magnetization curves of another 16 single magnetic nanoplatelets. Particles numbers are mentioned in the inset.

13. Magnetization as a function of the permanent magnet's position:

Fig. S8 shows magnetization curves of the four single magnetic nanoplatelets shown in Fig. 2 in the main text. Here, we plot the optical MCD signal as a function of the position of the permanent magnet from the sample, instead of the magnetic field. There is no change of noise amplitude on this plot, which demonstrates that the higher apparent noise at lower magnetic fields in the magnetization curves of Fig. 2 in the main text is an artefact. Because we measure with a constant stepping in magnet position, we obtain a much denser sampling of weak fields at large distances compared to the sampling of high fields at short distances (e.g. the field generated by a permanent magnetic is non-linear in its distance to it).

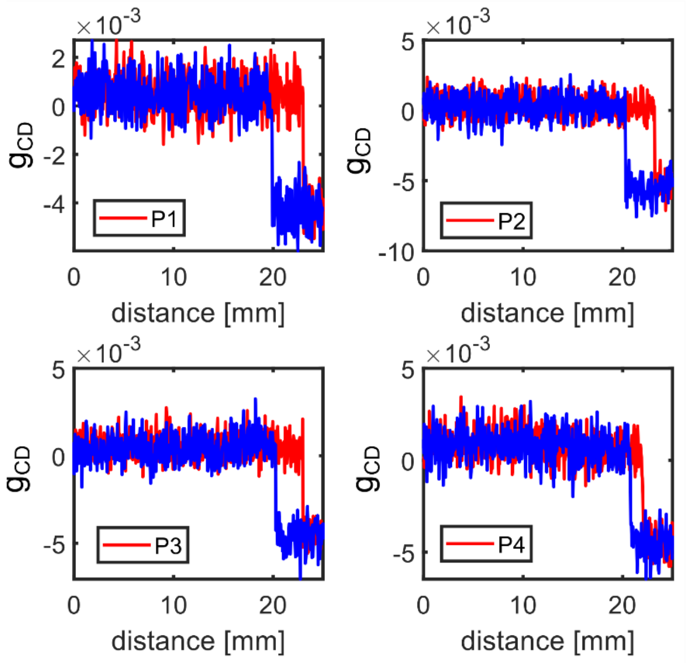

Figure. S8: Magnetization curves of the four nanoplatelets shown in Fig. 2 in the main text. Here, the MCD signal is plotted as a function of the distance of the magnet from the sample, only in the positive magnetic field direction.

14. MCD time traces at B = 0 and B = 280 mT:

Fig. S9 shows time traces of the MCD signal at B = 0 mT and B = 280 mT. Both time traces show similar noise levels. This again confirms that the higher noise level at lower fields apparent in Fig. 2 is only due to the larger number of data points at lower than at higher fields.

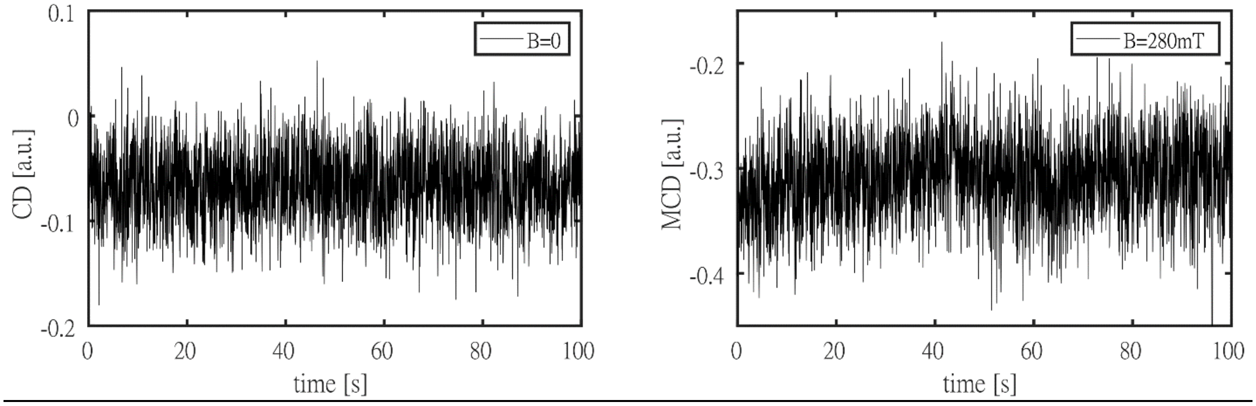

Figure. S9: (Left) CD time trace at B= 0 and (b) MCD time trace at B= 280 mT for a single magnetic platelet.

15. Histogram of on and off switching fields from time-dependent magnetization curve:

Fig. S10 shows the histogram of on and off switching fields of minor loops of a single NP measured over 15 sequential cycles as presented in Fig. 5 in the main text. The mean values and standard deviations of the histograms are mentioned in the inset. The distribution of on switching fields is broader than that of the off switching fields.

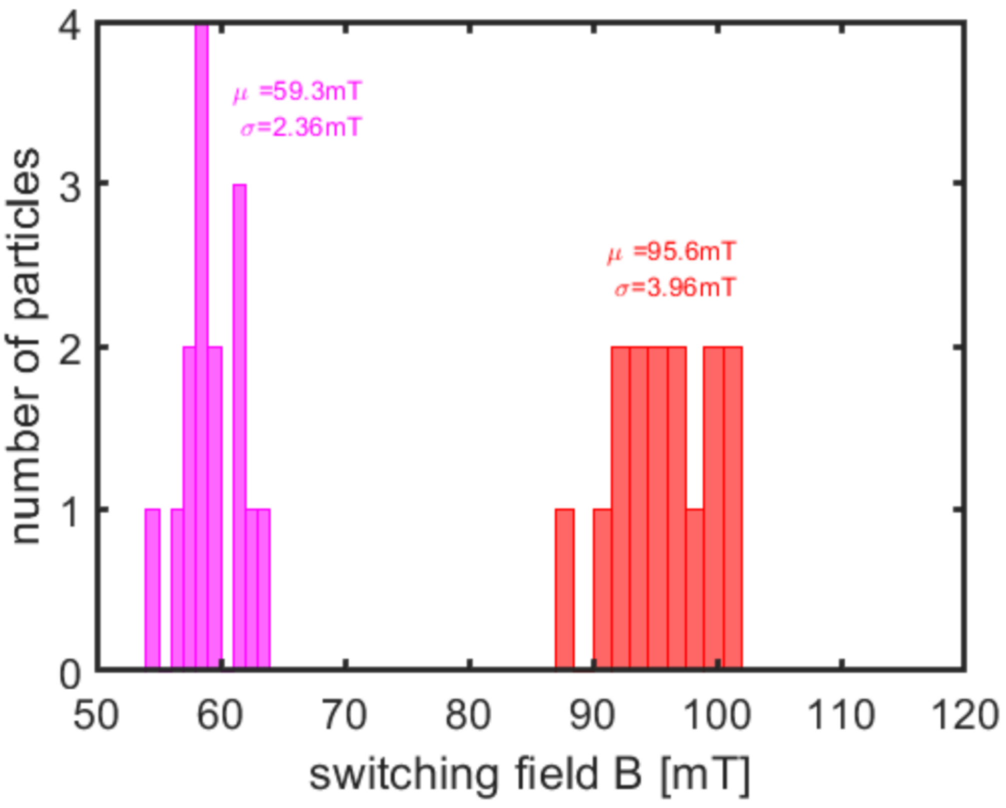

Figure S10: Histogram of off (magenta) and on (red) switching fields of minor loops of a single NP measured over 15 cycles as shown in Fig. 5 in the main text. The mean value (μ) and standard deviation (σ) is mentioned in the inset.

17. Simulation for calculating absorption of magnetic layers

An analytical calculation based on the transfer matrix method is used to estimate the optical absorption of each layer of the NPs.[3] The complex refractive indexes of different materials at the wavelength 532 nm, which is the wavelength used in PT MCD measurement, are used in the simulation.[4] Here we use the refractive index of Co to represent the CoB layer. In the simulation, we assume that the laser enters the platelet from the top side of the NP from air and has the following stack AIR/Ta(4)/Pt(2)/Co(0.8)/Pt(0.4)/Ru(0.8)/Pt(0.4)/Co(0.8)/Pt(2)/Ta(4)/AIR. The absorption of the laser of each layer is shown in Fig S11. From the estimation, only 8.7 % of the

light is absorbed in the two Co layers (4.5% in the first layer and 4.2% in the second layer) which will contribute to the MCD signal. Note that only 41.1% of the incoming light will be absorbed in the layer stack of the platelet (integrated absorption) as its thickness is less than the penetration depth of the light.

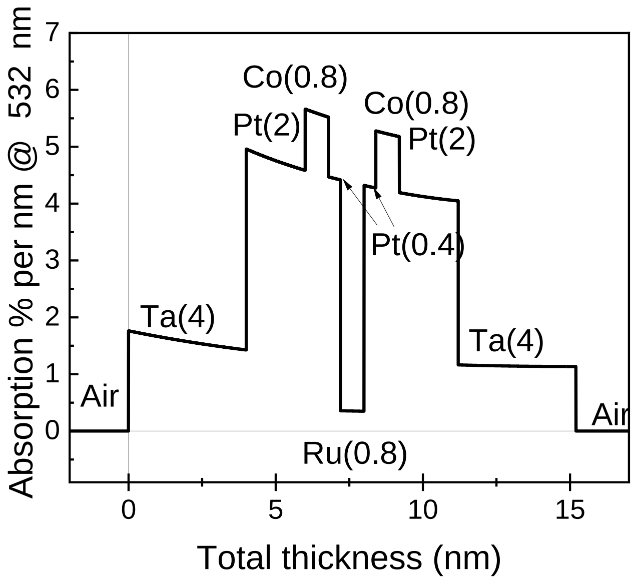

Figure S11: The absorption of the heating laser by the NP stack

18. The effect of dipolar field on the unreleased SAF NPs in SQUID measurement

As shown in Fig. S12, the unreleased SAF NPs are well aligned on the substrate with a constant distance of 335 nm between the two NPs. Dipolar fields from nearby NPs may influence the switching of NPs. When the external field is low, NPs are in the antiferromagnetic state leading to zero dipolar fields and thus not affecting the surrounding NPs. When NPs are saturated, the total dipolar fields at the central NP (in the red circle) are calculated as shown in Fig. S12. Note that we did not include NPs beyond 2 μm from the central NP, since their dipolar fields on the

position of the central NP are negligible. In addition, we calculate the field on the center of the central NPs. For a small area of the saturated NPs, the magnetic flux which is perpendicular to the substrate on the central NP is

$$\Delta B = \frac{\mu_0}{4\pi}\frac{\Delta m}{r^3} = \frac{\mu_0}{4\pi}\frac{M_s t \cdot \Delta area}{r^3}$$

where M_s is the saturation magnetization of CoB which is 1120 kA/m, *t* is the total thickness of the magnetic material which is 1.6 nm, *r* is the distance from the center of the central NP. By integrating on the area of all the NPs (except the central NP), we obtain a field of 0.5 mT. Considering the small value, we can neglect the effect of the dipolar field of neighboring NPs.

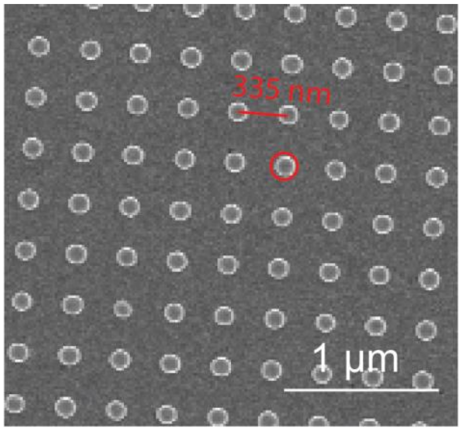

Figure S12: SEM of unreleased SAF NPs, which was used in SQUID.

<u>19. Schematic representation of the spin reversal mechanism</u>

The switching mechanism of ultrathin CoB PMA layers is known to start with the nucleation of small reversed areas[5]; these domains can then expand rapidly due to domain wall (~10 nm width in PMA systems) motion as the domain wall mobilty is high.[6] The initial nucleation process is a stochastic process and depends on the history and detailed morphology at the nanoscale for the

ultrathin CoB layer. Although the layers in the PMA-SAF stack are polycrystalline, the nucleation of the NPs is dominated by the strongest defect and our results show that this distribution is well defined. However to fully grasp the difference between the reversal of the on and off switch of the individual NP we first discuss the case of homogenous CoB layers in the NP and the effect of the generated dipole fields in the on and off state. Then we explain the effect of microstructure by including so-called nucleation embryos (strongest defects or hysteretic residuals of not fully reversed nanocrystals from the former saturation) where the stochastic domain nucleation happens and is followed by domain wall propagation.

The scheme in Fig. S13 represents the orientation of the magnetic moments in the two ferromagnetic layers of the nanoplatelet in the parallel (P) and anti-parallel (AP) states. Fig. S13 shows the ideal arrangements of the dipoles in the absence of any nucleation embryos. The same arrangement can be perturbed by the presence of some nucleation embryos. These schemes are meant to explain two observations on the minor hysteresis loop: (a) the much higher switching field in going from AP to P than when returning from P to AP; (b) the larger dispersion of switching fields when going from AP to P than when returning.

In the AP state, the generated dipole field of the two ferromagnetic layers has a component in the plane of the films which increases the switching probability at the edges of the NP. This will act as more active nucleation areas and hence the switching to the P arrangement is favored. In the inverse transition, when the field is lowered, the dipole field at the edges is collinear to the OOP direction. Hence, in this alignment there is no assistance of the dipolar field to the switching probability at the B_L (or B_{off}) switching field.

For the case of the same process in the presence of defects and nucleation embryos, the perturbations and stray fields created by these embryos will shift the switching fields B_{on} and B_{off} by random quantities, leading to a spread of switching fields when moving from particle to particle, or even, to a lesser extent, when repeating measurements on the same particle. Due to a lower sensitivity of the low switching field B_{off} to defects, the dispersion in this low switching field is lower.

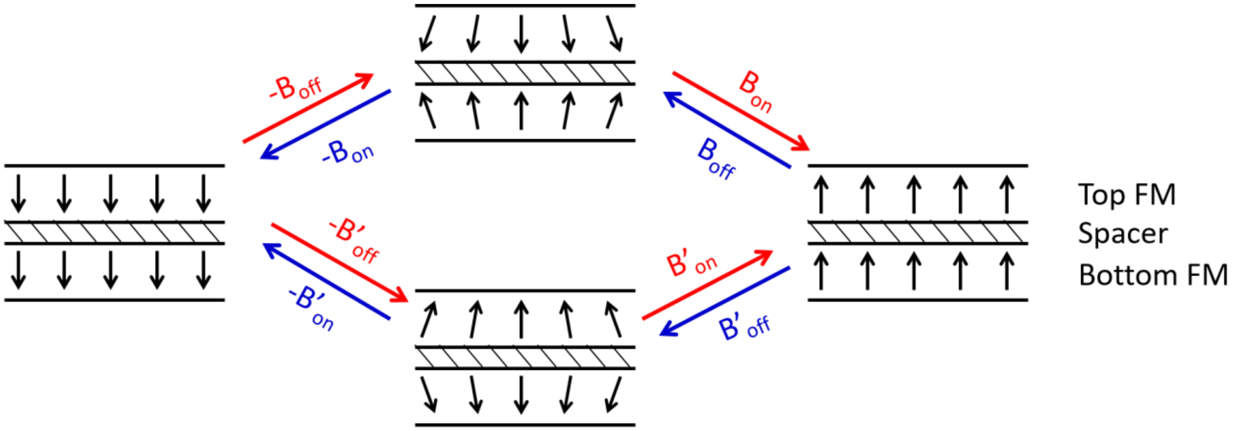

Figure S13: Two ferromagnetic layers antiferromagnetically coupled by the Ru spacer layer present anti-parallel (AP) states as represented in the middle and parallel (P) states as represented in the left and right. They are schematized in the absence of nucleation embryos and other defects. The red and blue color of the arrows representing the hysteresis loop of single particle PT MCD measurements. For the switching from the AP state to P state (B_{on}), the dipole field leads to canting of magnetization at the edge and assists the switching, whereas, for the switching from P state to AP state (B_{off}), the dipole fields are already aligned and do not contribute to the switching process.

REFERENCES

(1) Li, J.; van Nieuwkerk, P.; Verschuuren, M. A.; Koopmans, B.; Lavrijsen, R. Substrate Conformal Imprint Fabrication Process of Synthetic Antiferromagnetic Nanoplatelets. *arXiv:2206.15320v1* **2022**.

(2) Spaeth, P.; Adhikari, S.; Lahabi, K.; Baaske, M. D.; Wang, Y.; Orrit, M. Imaging the Magnetization of Single Magnetite Nanoparticle Clusters via Photothermal Circular Dichroism. *Nano Lett.* **2022**, *22* (9), 3645–3650. https://doi.org/10.1021/acs.nanolett.2c00178.

(3) Saleh, B.; Teich, M. *Fundamentals of Photonics, 3rd Edition*; 2019.

(4) Palik, E. D. *Handbook of Optical Constants of Solids*; Academic Press, 1998.

(5) Lavrijsen, R.; Malinowski, G.; Franken, J. H.; Kohlhepp, J. T.; Swagten, H. J. M.; Koopmans, B.; Czapkiewicz, M.; Stobiecki, T. Reduced Domain Wall Pinning in Ultrathin Pt/Co100−xBx/Pt with Perpendicular Magnetic Anisotropy. *Appl. Phys. Lett.* **2010**, *96* (2), 022501. https://doi.org/10.1063/1.3280373.

(6) Hu, G.; Thomson, T.; Rettner, C. T.; Raoux, S.; Terris, B. D. Magnetization Reversal in Co⁄Pd Nanostructures and Films. *Journal of Applied Physics* **2005**, *97* (10), 10J702. https://doi.org/10.1063/1.1849572.
